# Interpretable four-factor day-1 nomogram for predicting sepsis-associated encephalopathy in septic ICU patients with AKI: Development and internal validation in MIMIC-IV

**DOI:** 10.1097/MD.0000000000047726

**Published:** 2026-02-13

**Authors:** Zhiyang Zhang, Ze Zhang, Dandan Li, Li Guo, Heling Zhao, Limin Shen

**Affiliations:** a Department of Intensive Care Unit, Hebei General Hospital, Shijiazhuang City, China; b Department of Neonatal, Shijiazhuang Fourth Hospital, Shijiazhuang City, China.

**Keywords:** intensive care unit, machine learning, MIMIC-IV database, risk prediction model, sepsis-associated encephalopathy

## Abstract

Sepsis-associated encephalopathy (SAE) is common in the intensive care unit (ICU) and portends worse short- and long-term outcomes. To enable real-time bedside use and multicenter deployment, we aimed to develop a parsimonious, transparent day-1 prediction model using routinely available variables while preserving discrimination, calibration, and clinical utility. Using MIMIC-IV (2008–2022), we conducted a single-center retrospective study of adult sepsis patients with KDIGO-defined AKI. Predictors were restricted to the first 24 hours after ICU admission; the endpoint was any in-ICU SAE (“ever” vs “never”). After multiple imputation (m = 5), 44 baseline variables were standardized and entered into LASSO with 20-fold cross-validation. A 3-rule clinical screen (24 hours availability; non-treatment; low collinearity) distilled LASSO-selected features to a four-predictor logistic model; performance was internally validated (bootstrap) and compared with an XGBoost benchmark. SHAP analyses supported interpretability. Among 6780 ICU stays (training n = 4746; validation n = 2034), SAE occurred in 69.8%. The final 4 predictors were age, SAPS II, serum sodium, and mean arterial pressure (MAP). Discrimination was stable (AUC 0.734 training; 0.739 validation) with excellent calibration (validation CITL = −0.045; slope = 0.996; Brier = 0.182). Decision-curve analysis showed greater net benefit than XGBoost across thresholds 0.15 to 0.55; although AUCs were similar, XGBoost calibrated worse (CITL = −0.289; slope = 0.729). SHAP ranked contributions as SAPS II, sodium, age, and MAP, indicating a near-linear sodium-risk rise within 138 to 144 mmol/L, age-related risk above ~70 years, and a U-shaped MAP effect with protection around 55 to 75 mm Hg. We developed and validated a four-factor nomogram that uses only routine day-1 data to stratify SAE risk rapidly and transparently, outperforming a complex learner in calibration and net benefit. This parsimonious, interpretable tool highlights modifiable targets (sodium, individualized MAP) and provides a pragmatic foundation for multicenter validation and EMR-embedded early warning and intervention strategies.

## 1. Introduction

Sepsis is among the most devastating syndromes encountered in the intensive care unit (ICU), and its global incidence and burden continue to climb. Despite advances in care, in-hospital mortality persists at 25 to 40%, and survivors may endure months-to-years of physical debilitation, psychological disturbance, and cognitive decline.^[1]^ Beyond the systemic inflammatory storm and microcirculatory failure, injury to the central nervous system (CNS) critically shapes prognosis. Large cohort studies and systematic reviews show that 60 to 70% of patients develop sepsis-associated encephalopathy (SAE) in the acute phase. SAE is linked to higher in-hospital mortality, and survivors commonly exhibit persistent impairments in attention, executive function, and memory.^[2]^

Acute kidney injury (AKI) is another common complication of sepsis. A recent 2-center study, using the 2023 ADQI criteria, reported a SA-AKI incidence > 45% that independently predicted in-hospital death.^[3]^ After renal injury, uremic toxins, osmotic derangements, and inflammatory mediators cross the blood–brain barrier or activate glial cells, aggravating cerebral edema and microvascular dysfunction. Organ-interaction research has introduced the “kidney–brain axis,” whereby AKI releases indoxyl sulfate, IL-6, and other mediators that remotely drive neuroinflammation and precipitate SAE.^[4]^ Accordingly, multimodal strategies – precise fluid management, individualized blood-pressure and electrolyte targets – are becoming focal points in critical care.

Current SAE prediction tools are mostly single-center, retrospective models that require ≥ 10 clinical or inflammatory variables. For instance, Jin et al^[5]^ built a 20-variable nomogram in MIMIC-IV that achieved an AUC of ≈ 0.86. Its limitations are 2-fold. First, the large variable set necessitates costly assays (e.g., IL-6, procalcitonin). This requirement hampers real-time, low-burden bedside deployment. Second, excessive complexity delays multicenter implementation and evidence-based recalibration.

We aimed to develop and internally validate a parsimonious day-1 prognostic model for ICU-acquired SAE in septic patients with AKI and to benchmark it against a machine-learning approach. Leveraging MIMIC-IV, LASSO cross-validation, and clinical feasibility criteria, we screened > 40 variables and distilled 4 readily obtainable factors – age, SAPS II, serum sodium (Na^+^), and mean arterial pressure (MAP); SHAP then quantified contributions and highlighted a synergistic pattern consistent with kidney–brain dual-protection strategies, supporting future multicenter external validation.

## 2. Methods

### 2.1. Study design and data sources

This single-center, retrospective predictive-model study followed the TRIPOD reporting guidelines. Data were drawn from the MIMIC-IV v3.0 database (2008–2022, Beth Israel Deaconess Medical Center, Boston). The database had been reviewed and approved by the MIT-BIDMC dual institutional review boards, and all records are fully de-identified; individual informed consent was therefore waived.^[6]^ One of our team members completed the relevant courses of the Collaborative Institutional Training Initiative (CITI) and was granted database access (Certification Number: 60237018). Raw data were queried with SQL (Navicat, PremiumSoft CyberTech Ltd., Hong Kong, China); subsequent cleaning and analyses were carried out in R 4.4.1 (R Foundation, R Foundation for Statistical Computing, Vienna, Austria) and SPSS 26.0 (IBM, IBM Corp., Armonk).

### 2.2. Study population and endpoints

Inclusion criteria were 1) age ≥ 18 years; 2) ICU length of stay > 24 hours; 3) fulfillment of Sepsis-3^[7]^ and KDIGO-AKI^[8]^ diagnostic criteria at enrollment. Exclusion criteria (full ICD-9/10 codes in Table S1 [Supplemental Digital Content, https://links.lww.com/MD/R379]) comprised: primary brain injury; severe psychiatric or neurological disease; alcohol or substance abuse; metabolic or hepatic encephalopathy; severe electrolyte or glucose derangement; PaCO_2_ > 80 mm Hg; deep sedation on admission (RASS ≤ −4); major trauma or burns; recent cardiopulmonary resuscitation; and missing GCS or renal-function data.

The primary endpoint was sepsis-associated encephalopathy (SAE), defined as either a GCS < 15 at the first feasible neurological assessment or RASS ≥ −3 with a positive CAM-ICU delirium screen; SAE status was ascertained at any time during the ICU stay (“ever” vs “never”).^[9–11]^

Because precise SAE onset timestamps were unavailable, we prespecified a day-1 early risk-stratification design: candidate predictors were limited to 0 to 24 hours after ICU admission, while the outcome captured any subsequent in-ICU SAE (“ever” vs “never”). Patients unassessable on admission (e.g., RASS ≤ −4) were excluded to mitigate co-temporality.

No a priori sample size calculation was performed; all eligible ICU stays meeting Sepsis-3 and KDIGO-AKI criteria in MIMIC-IV (2008–2022) were included to maximize precision of model development and internal validation.

### 2.3. Variable collection and data preprocessing

All predictors were obtained within the first 24 hours after ICU admission comprised age, sex, and race. Vital signs included heart rate, mean arterial pressure (MAP), respiratory rate, body temperature, and peripheral oxygen saturation (SpO_2_). Laboratory data covered sodium (Na^+^), potassium (K^+^), serum creatinine (Scr), blood urea nitrogen (BUN), and coagulation indices. Illness severity was quantified with the SAPS II score. Therapy-related variables captured mechanical ventilation, renal-replacement therapy, sedation/analgesia use, and 24-h fluid balance (intake and output). We first evaluated the proportion of missing data. Variables missing <10% were retained (Table S2, Supplemental Digital Content, https://links.lww.com/MD/R379); those missing ≥10% were discarded.

### 2.4. Modeling process

We first split the 6780 eligible cases into a training cohort (n = 4746) and a validation cohort (n = 2034) using a 7:3 random allocation. Table 1 and Table S4 (Supplemental Digital Content, https://links.lww.com/MD/R379) summarize the baseline profiles and univariate logistic-regression outputs for all 44 candidate predictors; these statistics serve only descriptive and exploratory purposes and were not used for variable filtering so as to avoid a priori selection bias. Missing data were handled using MICE (*m* = 5). All modeling and validation were repeated in each imputed dataset and estimates were pooled using Rubin rules. Performance was consistent across imputations (Table S3, Supplemental Digital Content, https://links.lww.com/MD/R379). All reported statistics (OR, AUC, calibration metrics) are pooled across the 5 imputations. A sensitivity analysis confirmed that model robustness was not materially affected by imputation variance. Subsequently, all 44 predictors were z-standardized (mean = 0, SD = 1) to ensure scale comparability. The standardized variables were then entered en bloc into LASSO logistic regression (glmnet, α = 1). The λ_1_ₛₑ chosen via 20-fold cross-validation (the most parsimonious model within one standard error of the minimum deviance) yielded 18 non-zero coefficients. To ensure true bedside feasibility, we manually triaged the 18 features using 3 clinical rules: 1) obtainable within 24 hours of admission; 2) not a treatment-process variable; 3) physiologically and statistically independent of other features.^[12]^ Applying these rules retained 4 readily available predictors – age, SAPS II, serum sodium (Na^+^), and mean arterial pressure (MAP). The 4 predictors were refitted within each imputed dataset, and pooled estimates are reported below using unpenalized logistic regression. Variance-inflation factors ranged from 1.01 to 1.16, confirming negligible collinearity. To probe any added value from complex learners, we trained an XGBoost model on the same feature set as a benchmark. XGBoost used default hyperparameters and implemented 20-fold CV early-stopping on training-set AUC. Because nomograms apply only to parametric generalized-linear models, a nomogram was generated solely for the final logistic model.

**Table 1:** Baseline characteristics of patients with sepsis and AKI.

	All patients	Training cohort	SAE patients	*P*	All patients	Validation cohort	SAE patients	*P*
		Non-SAE patients				Non-SAE patients		
	N = 4746	N = 1432	N = 3314		N = 2034	N = 625	N = 1409	
Patient characteristics								
Age (yr)	70.23 (60.69, 80.76)	65.21 (56.00, 74.40)	72.49 (62.50, 82.50)	<.001	69.89 (59.25, 79.39)	64.50 (54.81, 74.32)	72.61 (62.11, 81.40)	<.001
Sex, n (%)								
Male	1939 (40.9)	490 (34.2)	1449 (43.7)	<.001	845 (41.5)	218 (34.9)	627 (44.5)	<.001
Female	2807 (59.1)	942 (65.8)	1865 (56.3)		1189 (58.5)	407 (65.1)	782 (55.5)	
Race (simplified), n (%)								
White	3104 (65.4)	969 (67.7)	2135 (64.4)	.017	1314 (64.6)	419 (67.0)	895 (63.5)	.076
B/A	360 (7.6)	89 (6.2)	271 (8.2)		157 (7.7)	40 (6.4)	117 (8.3)	
Other	839 (17.7)	258 (18.0)	581 (17.5)		342 (16.8)	111 (17.8)	231 (16.4)	
M/M	443 (9.3)	116 (8.1)	327 (9.9)		221 (10.9)	55 (8.8)	166 (11.8)	
First hospital stay, n (%)								
No	1186 (25.0)	306 (21.4)	880 (26.6)	<.001	450 (22.1)	121 (19.4)	329 (23.3)	.045
Yes	3560 (75.0)	1126 (78.6)	2434 (73.4)		1584 (77.9)	504 (80.6)	1080 (76.7)	
First ICU Stay, n (%)								
No	128 (2.7)	28 (2.0)	100 (3.0)	.038	52 (2.6)	11 (1.8)	41 (2.9)	.130
Yes	4618 (97.3)	1404 (98.0)	3214 (97.0)		1982 (97.4)	614 (98.2)	1368 (97.1)	
Hospital admission frequency, n (%)								
1	3560 (75)	1126 (78.6)	2434 (73.4)	<.001	1584 (77.9)	504 (80.6)	1080 (76.7)	.135
2	658 (13.9)	167 11.7)	491 (14.8)		261 (12.8)	70 (11.2)	191 (13.6)	
3 or more times	528 (11.1)	139 (9.7)	389 (11.8)		189 (9.3)	51 (8.2)	138 (9.7)	
AKI stage, n (%)								
1	1608 (33.9)	545 (38.1)	1063 (32.1)	<.001	722 (35.5)	241 (38.5)	481 (34.1)	.033
2	2370 (49.9)	691 (48.3)	1679 (50.7)		992 (48.8)	303 (48.5)	689 (48.9)	
3	768 (16.2)	196 (13.6)	572 (17.2)		320 (15.7)	81 (13.0)	239 (17.0)	
Vital signs								
Heart rate	86 (77, 101)	83 (75, 96)	87 (77, 102)	<.001	86 (76, 100)	84 (75, 96)	87 (77, 101)	<.001
Respiratory rate	18 (15, 22)	17 (14, 22)	18 (15, 23)	<.001	18 (15, 22)	17 (14, 22)	18 (15, 23)	<.001
SBP (mm Hg)	116 (102, 133)	114 (103, 129)	117 (102, 135)	.002	115 (102, 131)	114 (103, 129)	116 (102, 133)	.507
DBP (mm Hg)	63 (54, 74)	62 (54, 72)	63 (54, 75)	.004	63 (54, 74)	63 (54, 73)	63 (54, 74)	.862
MAP (mm Hg)	81 (71, 93)	80 (71, 90)	82 (71, 94)	<.001	81 (71, 92)	80 (71, 91)	81 (71, 92)	.402
Temperature (°C)	36.61 (36.33, 37.00)	36.56 (36.28, 36.94)	36.67 (36.33, 37.06)	<.001	36.61 (36.33, 37.00)	36.56 (36.28, 37.00)	36.67 (36.39, 37.00)	.005
SpO_2_ (%)	99 (95, 100)	99 (96, 100)	98 (95, 100)	<.001	99 (96, 100)	99 (96, 100)	98 (95, 100)	<.001
Comorbidities (n, %)								
Hypertension, n (%)								
No	1306 (27.5)	410 (28.6)	896 (27.0)	.259	622 (30.6)	180 (28.8)	442 (31.4)	.246
Yes	3440 (72.5)	1022 (71.4)	2418 (73.0)		1412 (69.4)	445 (71.2)	967 (68.6)	
Diabetes, n (%)								
No	3596 (75.8)	1111 (77.6)	2485 (75.0)	.055	1548 (76.1)	481 (77.0)	1067 (75.7)	.548
Yes	1150 (24.2)	321 (22.4)	829 (25.0)		486 (23.9)	144 (23.0)	342 (24.3)	
COPD, n (%)								
No	4012 (84.5)	1226 (85.6)	2786 (84.1)	.176	1711 (84.1)	536 (85.8)	1175 (83.4)	.178
Yes	734 (15.5)	206 (14.4)	528 (15.9)		323 (15.9)	89 (14.2)	234 (16.6)	
CKD, n (%)								
No	3440 (72.5)	1050 (73.3)	2390 (72.1)	.393	1513 (74.4)	464 (74.2)	1049 (74.4)	.920
Yes	1306 (27.5)	382 (26.7)	924 (27.9)		521 (25.6)	161 (25.8)	360 (25.6)	
Cardiovascular disease, n (%)								
No	264 (5.6)	79 (5.5)	185 (5.6)	.928	112 (5.5)	28 (4.5)	84 (6.0)	.177
Yes	4482 (94.4)	1353 (94.5)	3129 (94.4)		1922 (94.5)	597 (95.5)	1325 (94.0)	
Laboratory parameters								
BUN (mg/dL)	22 (15, 36)	20 (14, 32)	23 (16, 39)	<.001	22 (15, 37)	19 (15, 30)	24 (16, 40)	<.001
Chloride (mmol/L)	105 (101, 109)	106 (102, 110)	105 (101, 109)	.005	106 (101, 109)	106 (102, 110)	105 (101, 109)	.126
Scr (mg/dL)	1.1 (0.8, 1.8)	1.1 (0.8, 1.7)	1.1 (0.8, 1.8)	.12	1.1 (0.8, 1.7)	1.1 (0.8, 1.7)	1.1 (0.8, 1.7)	.271
Sodium (mmol/L)	139 (137, 141)	138 (136, 140)	139 (137, 142)	<.001	139 (137, 141)	138 (136, 140)	140 (137, 142)	<.001
Potassium (mmol/L)	4.2 (3.8, 4.6)	4.2 (3.9, 4.6)	4.2 (3.8, 4.6)	.012	4.2 (3.8, 4.6)	4.2 (3.9, 4.6)	4.2 (3.8, 4.6)	.427
INR	1 (1,2)	1 (1,2)	1 (1,2)	.07	1 (1,2)	1 (1,2)	1 (1,2)	.104
PT (s)	15 (13, 18)	15 (14, 17)	15 (13, 18)	.462	15 (14, 18)	15 (14, 17)	16 (14, 18)	.216
PTT (s)	33 (28, 39)	32 (28, 38)	33 (28, 39)	<.001	32 (28, 39)	32 (28, 38)	33 (28, 39)	.026
WBC (×10^9^/L)	11.9 (8.5, 16.5)	11.9 (8.5, 16.5)	12 (8.5, 16.5)	.917	12.0 (8.4, 16.4)	11.9 (7.9, 15.9)	12.1 (8.6, 16.6)	.054
Platelet (×10^9^/L)	176 (125, 241)	174 (128, 242)	177 (124, 240)	.396	174 (127, 242)	170 (128, 237)	176 (126, 244)	.671
Hemoglobin (g/L)	10.1 (8.6, 11.7)	10.0 (8.7, 11.5)	10.1 (8.6, 11.7)	.270	10.0 (8.6, 11.5)	10.0 (8.7, 11.5)	10.0 (8.5, 11.5)	.909
Magnesium (mmol/L)	2 (2,2)	2 (2,2)	2 (2,2)	.593	2 (2,2)	2 (2,2)	2 (2,2)	.669
SAPS II	40 (32, 50)	35 (29, 42)	43 (35, 53)	<.001	41 (33, 51)	35 (28, 42)	44 (35, 54)	<.001
Therapeutic interventions								
Ventilator, n (%)								
No	2057 (43.3)	696 (48.6)	1361 (41.4)	<.001	862 (42.4)	290 (46.4)	572 (40.6)	.015
Yes	2689 (56.7)	736 (51.4)	1953 (58.9)		1172 (57.6)	335 (53.6)	837 (59.4)	
RRT, n (%)								
No	4400 (92.7)	1357 (94.8)	3043 (91.8)	<.001	1898 (93.3)	595 (95.2)	1303 (92.5)	.023
Yes	346 (7.3)	75 (5.2)	271 (8.2)		136 (6.7)	30 (4.8)	106 (7.5)	
Sedative, n (%)								
No	1982 (41.8)	574 (40.1)	1408 (42.5)	.123	829 (40.8)	234 (37.4)	595 (42.2)	.043
Yes	2764 (58.2)	858 (59.9)	1906 (57.5)		1205 (59.2)	391 (62.6)	814 (57.8)	
Analgesic, n (%)								
No	2880 (60.7)	947 (66.1)	1933 (58.3)	<.001	1230 (60.5)	403 (64.5)	827 (58.7)	.014
Yes	1866 (39.3)	485 (33.9)	1381 (41.7)		804 (39.5)	222 (35.5)	582 (41.3)	
H_2_ Antagonist, n (%)								
No	3558 (75.0)	1122 (78.4)	2436 (73.5)	<.001	1500 (73.7)	484 (77.4)	1016 (72.1)	.012
Yes	1188 (25.0)	310 (21.6)	878 (26.5)		534 (26.3)	141 (22.6)	393 (27.9)	
Heparin, n (%)								
No	2912 (61.4)	971 (67.8)	1941 (58.6)	<.001	1264 (62.1)	446 (71.4)	818 (58.1)	<.001
Yes	1834 (38.6)	461 (32.2)	1373 (41.4)		770 (37.9)	179 (28.6)	591 (41.9)	
Diuretic, n (%)								
No	2410 (50.8)	783 (54.7)	1627 (49.1)	<.001	1020 (50.1)	326 (52.2)	694 (49.3)	.227
Yes	2336 (49.2)	649 (45.3)	1687 (50.9)		1014 (49.9)	299 (47.8)	715 (50.7)	
Vasoactive agent, n (%)								
No	2127 (44.8)	616 (43.0)	1511 (45.6)	.101	897 (44.1)	282 (45.1)	615 (43.6)	.537
Yes	2619 (55.2)	816 (57.0)	1803 (54.4)		1137 (55.9)	343 (54.9)	794 (56.4)	
Renal and ventilation parameters								
Total input, n (%)								
Low	1199 (25.3)	338 (23.6)	861 (26.0)	.126	496 (24.4)	142 (22.7)	354 (25.1)	.384
Medium	2374 (50.0)	746 (52.1)	1628 (49.1)		1017 (50.0)	313 (50.1)	704 (50.0)	
High	1173 (24.7)	348 (24.3)	825 (24.9)		521 (25.6)	170 (27.2)	351 (24.9)	
Total output, n (%)								
Low	1219 (25.7)	305 (21.3)	914 (27.6)	<.001	476 (23.4)	120 (19.2)	356 (25.3)	<.001
Medium	2365 (49.8)	679 (47.4)	1686 (50.9)		1025 (50.4)	304 (48.6)	721 (51.2)	
High	1162 (24.5)	448 (31.3)	714 (21.5)		533 (26.2)	201 (32.2)	332 (23.6)	
Fluid balance, n (%)								
Low	1204 (25.4)	345 (24.1)	859 (25.9)	.049	491 (24.1)	144 (23.0)	347 (24.6)	.695
Medium	2367 (49.9)	753 (52.6)	1614 (48.7)		1023 (50.3)	322 (51.5)	701 (49.8)	
High	1175 (24.8)	334 (23.3)	841 (25.4)		520 (25.6)	159 (25.5)	361 (25.6)	
Urine output, n (%)								
Low	1205 (25.4)	285 (19.9)	920 (27.8)	<.001	495 (24.4)	118 (18.9)	377 (26.8)	<.001
Medium	2392 (50.4)	720 (50.3)	1672 (50.5)		997 (49.0)	317 (50.7)	680 (48.2)	
High	1149 (24.2)	427 (29.8)	722 (21.7)		542 (26.6)	190 (30.4)	352 (25.0)	

Race (simplified): White = White or Caucasian; B/A = Black or African-American; other = Asian, Native American, Pacific Islander, or unknown race; M/M = mixed or multiple racial identities. Data are presented as median (IQR) or n (%). Total input, output, and net balance refer to the first 24 hours in the ICU and are categorized as: Low (<1500 mL), Medium (1500–2000 mL), and High (>2000 mL). Ventilation and RRT indicate initiation within the first 24 hours of ICU admission. Due to the large sample size, minor differences may reach statistical significance; however, most variables show standardized mean differences ( SMD ) < 0.10. *P* values are exploratory and were not adjusted for multiple comparisons.

AKI = acute kidney injury, BUN = blood urea nitrogen, CKD = chronic kidney disease, COPD = chronic obstructive pulmonary disease, DBP = diastolic blood pressure, INR = international normalized ratio, MAP = mean arterial pressure, PT = prothrombin time, PTT = partial thromboplastin time, RRT = renal-replacement therapy, SAE = sepsis-associated encephalopathy, SAPS II = Simplified Acute Physiology Score II, SBP = systolic blood pressure, Scr = serum creatinine, SpO_2_ = peripheral oxygen saturation, WBC = white blood cells.

For bedside interpretability, effect sizes are presented per clinically meaningful increments (age = 10 years, MAP = 5 mm Hg, sodium = 5 mmol/L, SAPS II = 10 points); regression coefficients are estimated on the original clinical scales.

### 2.5. Statistical analysis and model evaluation

Variable normality was assessed with the Shapiro–Wilk test. Normally distributed variables were summarized as mean ± standard deviation and compared with the independent-samples *t* test. Non-normal variables were expressed as median (interquartile range) and compared using the Mann–Whitney *U* test. Categorical variables were reported as counts (percentages) and analyzed with the χ^2^ test or Fisher exact test, as appropriate. Discrimination was quantified by the area under the receiver-operating-characteristic curve (AUC) with 95% confidence intervals. Calibration was assessed using calibration plots, calibration-in-the-large (CITL), calibration slope, Brier score, and the Hosmer–Lemeshow test. Clinical utility was quantified by decision-curve analysis over threshold probabilities 0.15 to 0.55. Interpretability (for the machine-learning model) used DALEX to compute SHAP values, global importance ranks, dependence plots, and waterfall plots. All analyses were repeated within each imputed dataset (*m* = 5), bootstrap-corrected (*B* = 1000) per imputation, and estimates were pooled using Rubin rules. All hypothesis tests were 2-tailed (*P* < .05), and all reported statistics (OR, AUC, calibration metrics) are pooled across the 5 imputations. As the outcome (“any in-ICU SAE”) was ascertained during the index ICU stay, loss to follow-up was not applicable.

### 2.6. Software and reproducibility

Key R packages included tidyverse 2.0, glmnet 4.1-8, pROC, rmda, DALEX, MICE, and related dependencies. All analysis scripts used for data extraction, modeling, and figure generation are available at GitHub. Due to data sharing restrictions, no patient-level data or pseudo-datasets are provided.

## 3. Result

### 3.1. Patient selection and baseline characteristics

**Figure 1.**
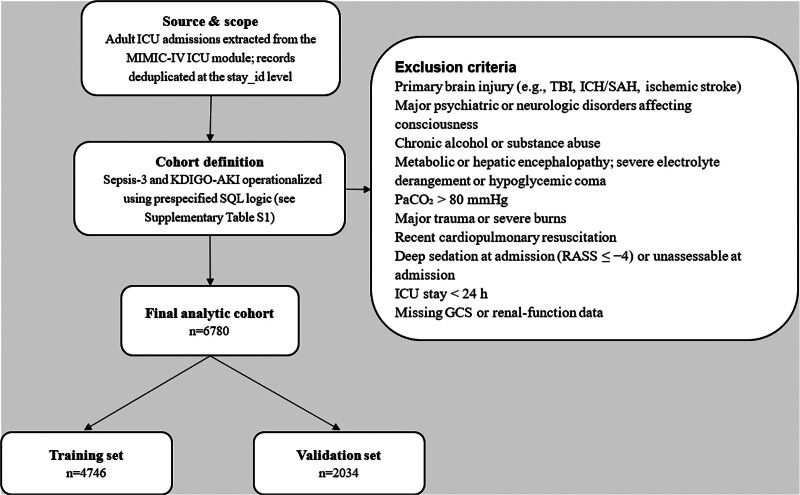
Cohort construction. Adult ICU admissions from the MIMIC-IV ICU module (de-duplicated at stay_id) were screened; Sepsis-3 and KDIGO-AKI were implemented via prespecified SQL (Table S1, Supplemental Digital Content, https://links.lww.com/MD/R379). Exclusions are listed; the final analytic cohort was n = 6780, split 7:3 into training (n = 4746) and validation (n = 2034). AKI = acute kidney injury, ICU = intensive care unit, KDIGO = Kidney Disease: Improving Global Outcomes, MIMIC-IV = Medical Information Mart for Intensive Care IV.

Figure 1 illustrates the cohort construction: adult ICU stays from MIMIC-IV were screened using prespecified Sepsis-3 and KDIGO-AKI logic (see Table S1, Supplemental Digital Content, https://links.lww.com/MD/R379). After excluding records with primary brain injury or other consciousness-affecting neurologic/psychiatric disorders, severe metabolic/hepatic encephalopathy or electrolyte/glucose derangements, PaCO_2_ > 80 mm Hg, recent cardiopulmonary resuscitation, deep sedation at admission (RASS ≤ −4) or unassessable neurological status, ICU stay < 24 hours, and missing key data, the final analytic cohort comprised 6780 stays. These were then randomly split 7:3 into a training set (n = 4746) and a held-out internal validation set (n = 2034). Baseline demographics and clinical variables were well balanced; >90% of variables showed an absolute standardized mean difference < 0.10. The overall incidence of SAE was 69.8%. Within the training cohort, SAE cases were older – median 72.5 years (IQR 62.5–82.5) vs 65.2 years (56.0–74.4) – and had higher SAPS II scores – 43 (35–53) vs 35 (29–42) (all *P* < .001). They also displayed mildly higher serum sodium – 139 mmol/L (136–143) vs 138 mmol/L (135–141) – and higher mean arterial pressure – 82 mm Hg (71–94) vs 80 mm Hg (71–90) (both *P *< .001). Rates of KDIGO stage 3 AKI (17.2% vs 10.3%), mechanical ventilation (58.9% vs 42.1%), and renal-replacement therapy (8.2% vs 4.6%) were likewise higher in the SAE group (all *P* < .001). The validation cohort mirrored these trends, confirming sampling reliability; full characteristics are provided in Table 1.

### 3.2. Univariate analysis and variable selection

**Figure 2.**
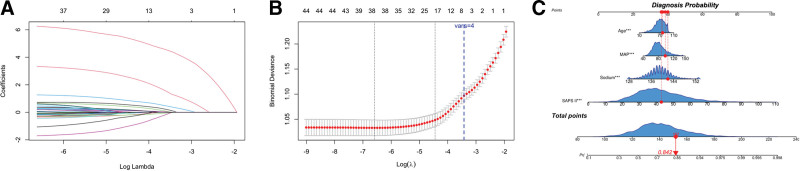
Feature selection and final model visualization. (A) LASSO coefficient paths for 44 baseline variables under 20-fold cross-validation; each curve shows the shrinkage trajectory as log(λ) increases. (B) 20-fold cross-validated binomial deviance versus log(λ) (lower is better). The left gray dashed line marks λ_min (minimum mean deviance), and the right gray dashed line marks λ_1_ₛₑ (largest λ within 1 SE of the minimum; 18 non-zero coefficients at this point; counts shown above the curve). The blue dashed line is a reference for the final four-predictor set obtained after applying 3 prespecified clinical rules (24-h bedside availability, non-treatment predictors, and low collinearity/stability); it is not a glmnet solution. The 4 predictors were subsequently refit with an unpenalized logistic regression. (C) Nomogram from the final logistic model (age, SAPS II, sodium, MAP). Red dots illustrate point assignment; the total points map to a demonstration probability. LASSO = least absolute shrinkage and selection operator, MAP = mean arterial pressure, SAPS II = Simplified Acute Physiology Score II.

Univariate logistic regression was applied to the 44 candidate variables in the training cohort (see Table S4, Supplemental Digital Content, https://links.lww.com/MD/R379). Twenty-four variables showed a significant association with SAE (*P* < .05). Age increased SAE risk (OR = 1.03 per year, 95% CI 1.03–1.04). Male sex conferred a higher risk (OR = 1.49, 95% CI = 1.32–1.68). Each additional SAPS II point raised the odds of SAE (OR = 1.07, 95% CI = 1.06–1.08). Serum sodium was positively associated (OR = 1.10 per mmol/L, 95% CI = 1.07–1.13). Blood urea nitrogen (BUN) showed a modest positive effect (OR = 1.02 per mmol/L, 95% CI = 1.01–1.03). Higher mean arterial pressure showed a modest positive association with SAE in univariate analysis (OR = 1.007 per mm Hg, 95% CI = 1.004–1.011). Greater negative fluid balance, early mechanical ventilation (OR = 0.74, 95% CI = 0.66–0.82), and renal-replacement therapy (OR = 0.62, 95% CI = 0.49–0.79) were likewise associated with lower SAE risk. To improve parsimony and bedside utility, we applied LASSO with 20-fold cross-validation. Figure 2A illustrates the LASSO coefficient shrinkage path across varying log(λ) values. At λ_1_ₛₑ, the model retained 18 features with non-zero coefficients. Applying 3 clinical filters – 24-h availability, non-treatment status, and physiological/statistical independence – further distilled the list. The final multivariable logistic model, therefore, incorporated 4 predictors: age, SAPS II, serum sodium, and MAP (Fig. 2B, blue dashed line). Variance-inflation factors (1.01–1.16) confirmed negligible multicollinearity.

### 3.3. Multi-factor model construction and validation

**Figure 3.**
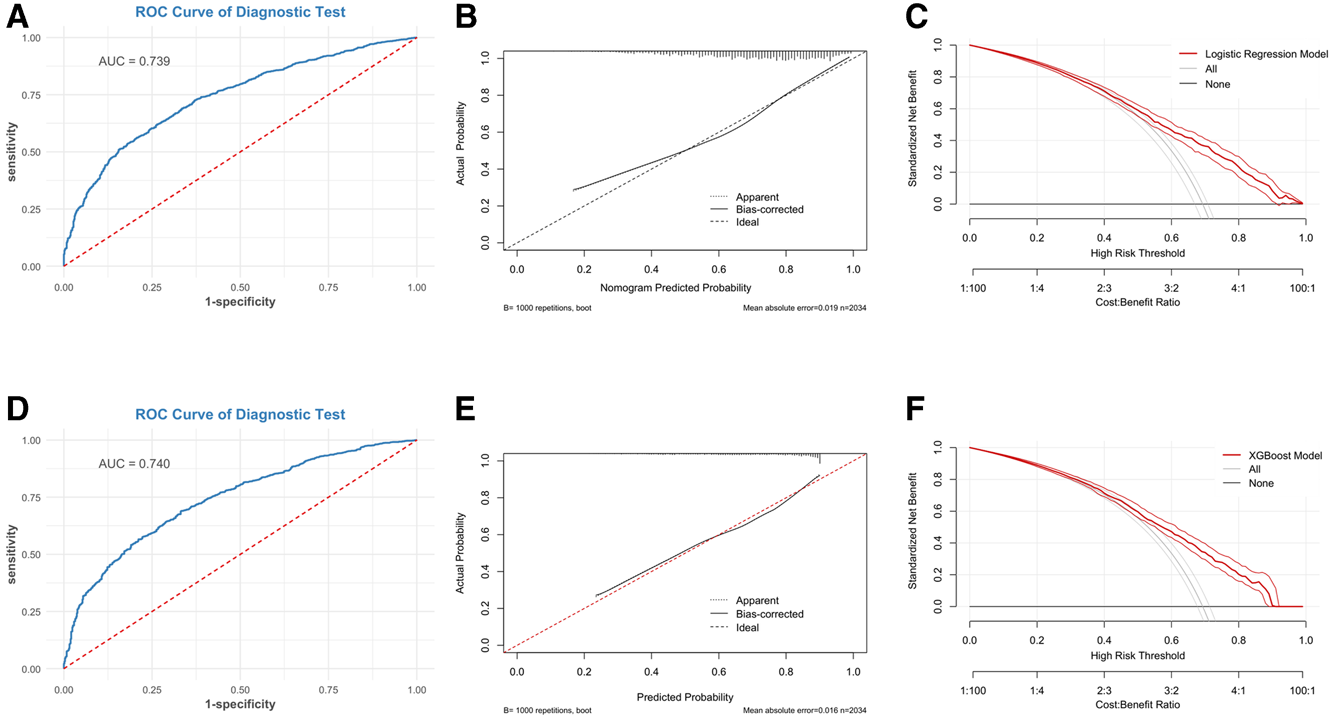
Early risk stratification of any in-ICU SAE using 0–24 hours data. ROC (A and D), calibration with CITL/slope (B and E), and decision curves (C and F). Metrics were internally validated (bootstrap) and pooled across *m* = 5 imputations. (A–C) Logistic regression model: (A) ROC curve showing an AUC of 0.739. (B) Calibration curve demonstrating good agreement between predicted and observed probabilities (bias-corrected line close to ideal). (C) Decision-curve analysis indicating clinical net benefit across a wide range of risk thresholds. (D–F) XGBoost model (for robustness comparison): (D) ROC curve with slightly improved AUC of 0.740. (E) Calibration curve showing systematic underestimation with over-dispersion (CITL − 0.289, slope 0.729). (F) Decision curve confirming comparable or better net benefit. Curves are averaged across 5 imputed validation sets. Training-set counterparts are shown in Figure S1 (Supplemental Digital Content, https://links.lww.com/MD/R379). AUC = area under the receiver operating characteristic curve, CITL = calibration-in-the-large, ICU = intensive care unit, ROC = receiver operating characteristic, SAE = sepsis-associated encephalopathy.

Multivariable logistic regression confirmed that all 4 predictors were independently associated with SAE (Table 2). Reported per clinically meaningful increments, the odds of SAE increased by 82% per 10-point increase in SAPS II (OR = 1.825, 95% CI = 1.708–1.931), 63% per 5 mmol/L increase in serum sodium (OR = 1.625, 95% CI = 1.490–1.778), 13% per 10-year increase in age (OR = 1.127, 95% CI = 1.072–1.184), and 7% per 5-mm Hg increase in MAP (OR = 1.067, 95% CI = 1.046–1.093); all *P* < .001. Pooled across the 5 imputations, the nomogram achieved an AUC of 0.734 (95% CI = 0.721–0.747) in the training cohort and 0.739 (95% CI = 0.719–0.758) in the validation cohort, demonstrating robust discrimination. The corresponding nomogram is depicted in Figure 2C, which translates the 4 predictors into a visual bedside scoring tool. Calibration was excellent. In the training set, CITL ≈ 0 and slope ≈ 1.00 indicated minimal systematic bias, and the Brier score was 0.182. Although the large sample size yielded a significant Hosmer–Lemeshow statistic (χ^2^ = 34.8, *P* < .001), the calibration plot showed only minor group-level deviation (Fig. S1B, Supplemental Digital Content, https://links.lww.com/MD/R379). In the validation set, CITL = −0.045, slope = 0.996, and Brier = 0.182; the Hosmer–Lemeshow test showed no evidence of miscalibration (*P* = .16; see Fig. 3A–C and Fig. S1A–C, Supplemental Digital Content, https://links.lww.com/MD/R379.

**Table 2:** Multivariable logistic regression analysis of SAE risk factors.

Variable	β	Odds ratio (95% CI)	*P*
Sodium (mmol/L)	0.097	1.625 (1.490–1.778)	<.001
MAP (mm Hg)	0.013	1.067 (1.046–1.093)	<.001
Age (yr)	0.012	1.127 (1.072–1.184)	<.001
SAPS II	0.060	1.825 (1.708–1.931)	<.001

Odds ratios (ORs) are reported per clinically meaningful increments: Age per 10 years; MAP per 5 mm Hg; Sodium per 5 mmol/L; SAPS II per 10 points. Coefficients (β) are per 1 unit in original scales. The intercept (β_0_) and the full equation are provided in Table S5 (Supplemental Digital Content, https://links.lww.com/MD/R379).

MAP = mean arterial pressure, SAPS II = Simplified Acute Physiology Score II, Sodium = serum sodium concentration.

### 3.4. XGBoost algorithm comparison analysis

Pooled across the 5 imputations, XGBoost in the training cohort showed a higher apparent AUC of 0.782, yet calibration deviated from ideal (CITL = 0.001, slope = 1.252; Hosmer–Lemeshow *P* = .00068). In the held-out internal validation cohort, both models yielded similar AUCs (0.740 vs 0.739). However, XGBoost displayed systematic underestimation with over-dispersion (CITL = −0.289, slope = 0.729; HL *P* < .001), demonstrating substantially poorer calibration than the logistic model. Decision-curve analysis further showed that within the usual threshold range (0.15–0.55), the logistic nomogram consistently conferred greater net clinical benefit than XGBoost. Together, these findings underscore the four-factor logistic model’s superiority in discrimination, calibration, and bedside applicability (Fig. 3D–F and Fig. S1D–F, Supplemental Digital Content, https://links.lww.com/MD/R379).

### 3.5. Model interpretability analysis (SHAP analysis)

**Figure 4.**
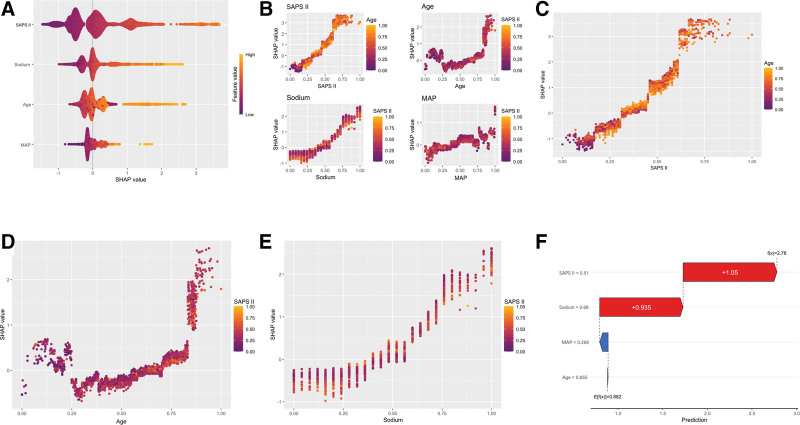
SHAP-based interpretation of the XGBoost model. (A) Summary plot of SHAP values ranked by feature importance, showing both the magnitude and direction of each variable’s impact. (B) SHAP dependence plots demonstrating the nonlinear contributions and interactions of SAPS II, age, sodium, and MAP. (C–E) Univariate SHAP profiles highlighting the individualized effect of SAPS II (C), age (D), and sodium (E) across the dataset, colored by SAPS II to show potential effect modification. (F) SHAP waterfall plot illustrating the additive contribution of each predictor to the final risk prediction in a representative patient. MAP = mean arterial pressure, SAPS II = Simplified Acute Physiology Score II, SHAP = Shapley additive explanations.

SHAP summary plots (Fig. 4A) ranked variables in descending contribution as SAPS II, serum sodium, age, and MAP, highlighting overall illness burden and osmotic stress as leading drivers of SAE risk. Partial-dependence curves showed a sharp risk inflection once SAPS II exceeded 42 points (standardized value ≈ 0.40); SAE risk rose almost linearly when serum sodium entered 138 to 144 mmol/L, accumulated markedly beyond 70 years of age, and displayed a U-shape for MAP – with protection between 55 and 75 mm Hg but higher risk below 55 mm Hg or above 90 mm Hg. An individual waterfall plot (Fig. 4F) corroborated these trends, illustrating how high illness severity, mild hypernatremia, advanced age, and out-of-range MAP synergistically pushed a representative patient across the SAE threshold. These interpretability findings suggest that early bedside interventions should prioritize hemodynamic stabilization and electrolyte correction within kidney–brain dual-protection bundles. Supplementary partial-dependence plots (Fig. 4B–E) and supplementary visualizations (Fig. S2A–D, Supplemental Digital Content, https://links.lww.com/MD/R379) provide further insights into the nonlinear and individualized effects of each predictor.

## 4. Discussion

### 4.1. Summary of key findings

We analyzed 6780 ICU admissions that simultaneously satisfied the Sepsis-3 and KDIGO criteria. To our knowledge, this is the first study to predict SAE using only 4 readily obtainable variables – age, SAPS II, serum sodium, and MAP. The resulting model achieved AUCs of 0.734 in the derivation cohort and 0.739 in the independent validation cohort. Calibration was excellent, with an intercept ≈ 0, a slope ≈ 1, and a Brier score of 0.182. Decision-curve analysis demonstrated the greatest net clinical benefit across the threshold range 0.15 to 0.55. Under identical conditions, the more complex XGBoost algorithm conferred no additional advantage. SHAP interpretability confirmed that disease severity, mild hypernatremia, advanced age, and out-of-range MAP excursions synergistically elevate risk. These insights furnish a physiological rationale for targeted optimization of hemodynamics and electrolyte balance. Because the nomogram relies solely on routinely collected data from the first 24 hours of ICU stay, its calculation takes < 1 min. Such efficiency allows seamless integration into electronic medical record systems for real-time alerting. As highlighted by recent systematic reviews,^[13]^ the model satisfies current calls for scalable, easily deployable prediction tools. Consequently, the four-factor nomogram can flag almost 70% of SAE events within the first ICU day. This capability lays an evidence-based foundation for prospective trials of sodium control and individualized MAP targets.

### 4.2. Comparison with previous studies

#### 4.2.1. Incidence and parsimony benefits

In our cohort, the SAE rate reached 69.8%, aligning with the 60–70% reported by contemporary MIMIC-IV and multicenter studies. This concordance underscores the substantial neurological burden carried by septic patients with concurrent AKI.^[14]^ Earlier prediction tools typically incorporated ≥ 10 clinical or laboratory markers,^[5]^ inflating bedside testing cost and integration complexity. Some models even rely on cerebral oxygenation indices or inflammatory biomarkers,^[15]^ further hampering point-of-care deployment. By contrast, our nomogram deploys just 4 readily obtainable variables yet preserves robust performance (AUC ≈ 0.74) and excellent calibration on external validation. These data substantiate the “simple-but-accurate” paradigm and support its broader transferability.

#### 4.2.2. Sodium and MAP thresholds, clinically verified

Our model shows that even mild hypernatremia (> 138 mmol/L) drives a near-linear risk increase, mirroring the “osmolar imbalance → BBB disruption → SAE” cascade reported in contemporary cohorts.^[16,17]^ A recent MIMIC-IV analysis likewise revealed a U-shaped association between serum osmolality and ICU delirium, identifying 286 to 301 mmol/L as the nadir of risk.^[18]^ Cerebral-autoregulation research consistently indicates that sustaining MAP near 60 to 75 mm Hg – while keeping short-term variability minimal – minimizes neurological risk. Conversely, MAP excursions beyond the upper (ULA) or lower (LLA) autoregulatory bounds – particularly when CV-MAP > 10% – are associated with markedly increased SAE or delirium incidence.^[19,20]^ Collectively, these data advocate a “brain–kidney dual-protection” strategy instead of a one-size-fits-all 65 mm Hg target for septic AKI.

#### 4.2.3. Predictive value of SAPS II

Recent evidence indicates that SAPS II discriminates ICU mortality substantially better than SOFA and remains broadly comparable to APACHE II.^[21,22]^ Acknowledging SAE’s tight link to overall illness burden, we selected SAPS II as the anchor predictor and used SHAP analysis to determine its largest marginal effect (inflection ≈ 42 points), thereby reinforcing SAPS II’s primacy in SAE risk stratification and neuro-prognostication.

### 4.3. Pathophysiology and mechanistic explanation

Sepsis-associated encephalopathy (SAE) arises from a synergy of blood–brain barrier (BBB) disruption, neuroinflammation, microcirculatory failure, and intrinsic neuronal vulnerability. SAPS II serves as a surrogate for systemic inflammation and multi-organ failure load. Excess inflammatory mediators, together with endothelial injury, weaken the BBB, precipitating cerebral edema and over-activation of glial cells.^[23,24]^ In our cohort, SHAP analysis showed that risk rose almost linearly once serum sodium reached 138 to 144 mmol/L. Hyperosmolar stress down-regulates tight-junction proteins and amplifies oxidative stress, thereby fueling the neuro-inflammatory cascade. This pattern parallels cohort data demonstrating a U-shaped relationship between serum osmolality and ICU delirium or SAE mortality.^[18,25]^ We used SAPS II as the single global severity indicator because it had lower missingness in our cohort and was retained by LASSO with strong information contribution, enabling a parsimonious day-1 tool. Including multiple severity scores simultaneously (e.g., SOFA/APACHE II) risks redundancy and construct overlap; prioritizing parsimony is consistent with TRIPOD guidance and facilitates bedside deployment.

Mean arterial pressure (MAP) exerts a biphasic influence. MAP < 55mmHg compromises cerebral perfusion and depletes mitochondrial energy, whereas MAP > 90 mm Hg predisposes to vasogenic edema and shear injury. Both the OPT PRESS randomized trial and large MIMIC-IV/eICU cohorts showed that maintaining MAP around 70 to 80 mm Hg markedly lowers 28-day mortality and acute brain dysfunction.^[26,27]^ The optimum 55 to 75 mm Hg window identified here sits at the lower edge of that safety band and dovetails with the “MAPopt” concept of personalized cerebral autoregulation. Advanced age magnifies these hemodynamic and osmotic risks. Two large elderly-ICU cohorts reported significantly higher SAE incidence and 1-year mortality in patients ≥ 70 years, with age itself remaining an independent risk factor.^[28,29]^ Experimental and translational work indicates that aging increases BBB permeability, shifts cerebrovascular autoregulation leftwards, and induces an “inflamm-aging” milieu. These changes collectively lower the perfusion and inflammatory-tolerance thresholds. Such mechanisms may explain the pronounced susceptibility of older patients to SAE. Prospective studies are required to confirm this hypothesis.

Taken together, substantial illness burden, mild hypernatremia, advanced age, and MAP excursions synergistically potentiate the “BBB disruption → neuro-inflammation → cerebral-perfusion imbalance” axis that propels SAE. Accordingly, frontline management should first rectify serum sodium and hemodynamic derangements to break this harmful positive-feedback loop.

### 4.4. Clinical significance and potential interventions

#### 4.4.1. Bedside rapid stratification and resource optimization

The four-factor nomogram draws solely on routine vital signs and laboratory results obtained within the first 24 hours of ICU admission, and it generates a risk estimate in under 1 minute. Embedding the algorithm into electronic-medical-record trigger rules enables real-time SAE alerts at the bedside. By flagging nearly 70% of high-risk patients in advance, the tool creates a window for EEG surveillance, cerebral oximetry, and titration of sedation depth. These features accord with a tiered-care philosophy.

#### 4.4.2. Individualized MAP management

Both SHAP attribution and decision-curve analyses converged on 55–75 mm Hg as the safest cerebral-perfusion band, lower than that required by some hypertensive patients yet high enough to avoid vasogenic edema from over-pressurization. Current Surviving Sepsis Campaign guidelines offer only a universal starting threshold of 65 mm Hg.^[29]^ Coupling autoregulatory monitoring with MAPopt evidence^[19,27,30]^ supports a refined, brain–kidney dual-protection target in septic patients with AKI.

#### 4.4.3. Manageable risks of mild hypernatremia

Even a mild rise in serum sodium (138 to 144 mmol/L) markedly increases SAE risk. ICU-acquired hypernatremia is independently linked to 28-day mortality and delirium.^[17]^ In a recent cohort of 4265 patients with severe hypernatremia, lowering sodium by > 10 mmol/L within 24 hours (mean rate > 0.5 mmol/L/h) reduced 30-day and 1-year mortality without neurological complications.^[31]^ Accordingly, early correction of ICU-acquired hypernatremia and avoidance of prolonged hyperosmolarity may outperform the traditional “slow-correction” paradigm in SAE-prone patients. Prospective studies should test whether judicious reduction of crystalloid sodium or timely renal-replacement therapy can expedite natriuresis.

#### 4.4.4. Comprehensive management from a kidney–brain axis perspective

AKI and sepsis-associated encephalopathy share common inflammatory, endothelial, and microcirculatory injury pathways. By integrating serum sodium and MAP, our model balances the competing physiological needs of both organs. Future closed-loop systems that merge real-time renal function and cerebral-flow monitoring could dynamically titrate these targets. Such technology would operationalize an evidence-based “kidney–brain dual-protection” strategy.

### 4.5. Research advantages and innovation

Our study strikes a pragmatic balance between data depth, methodological transparency, and bedside feasibility, evidenced in 4 domains: (1) Large, well-defined ICU cohort: Adult ICU stays in MIMIC-IV were screened using prespecified Sepsis-3 and KDIGO-AKI logic. After applying the prespecified exclusion criteria, 6780 sepsis-AKI stays formed the analytic cohort. We then performed a stratified 7:3 random split to create a training and a held-out internal validation set, and used 20-fold cross-validation during model development to reduce resampling bias. (2) Simple-yet-accurate variable strategy: LASSO-CV shrank the candidate set to 18 variables at λ_1_ₛₑ; applying 3 pragmatic principles – immediate availability, treatment independence, and physiological orthogonality – distilled them to 4 factors. This parsimony preserved discrimination and calibration while maximizing bedside utility. (3) Full-process, multidimensional validation: We report AUC, calibration intercept and slope, Brier score, Hosmer–Lemeshow test, decision-curve analysis, and SHAP explanations. These metrics accord with TRIPOD and next-generation AI/ML transparency guidance, bolstering reproducibility and credibility.^[32]^ (4) Interpretability over complexity: Compared with XGBoost, the four-factor logistic model shows that greater algorithmic complexity is not inherently superior. The simpler model retained robust internal calibration and delivered higher net benefit, underscoring the enduring value of traditional methods when interpretability is paramount. Collectively, these strengths lay a firm groundwork for multicenter validation and prospective interventional trials.

### 4.6. Limitations

Despite multiple methodological safeguards, several limitations warrant cautious interpretation. First, this is a single-center, retrospective analysis based solely on the BIDMC subset of MIMIC-IV; center-specific practice patterns and the absence of external validation may restrict generalizability despite internal bootstrap validation. Second (temporal alignment), the primary endpoint was defined as the first occurrence of SAE at any time during the ICU stay (“ever” vs “never”), while predictors were restricted to measurements obtained within 0 to 24 hours after ICU admission. Because exact onset timestamps of SAE were not consistently available, the present work should be interpreted as day-1 early risk stratification rather than strict prospective prediction; some co-temporality is possible for very early events, although we excluded patients unassessable at admission (e.g., RASS ≤ −4) to mitigate this issue. Third, residual confounding may persist because key variables – such as time-varying sedation depth/exposure, the timing and adequacy of infection control, or dialysate sodium – were incompletely captured; in addition, SAE ascertainment may be affected by the frequency and feasibility of GCS/CAM-ICU assessments under sedation or procedures. Fourth, missing data were modest for each predictor (<10%) and were handled using multiple imputation by chained equations (m = 5); nevertheless, the MAR assumption is untestable and violations could bias estimates despite our pragmatic approach. Fifth, the model relies on static first-day variables and omits high-resolution time-series and time-to-event/competing-risk modeling, which may underrepresent dynamic physiology and discharge/death as competing outcomes. Sixth, ICD-9/10-based exclusion criteria for primary brain injury and related conditions are susceptible to coding variability and misclassification. Finally, no intervention-level evidence is provided: decision-curve analysis quantifies theoretical net benefit only, and prospective, multicenter studies – ideally with reliable SAE onset timestamps, dynamic predictors, and pragmatic impact evaluations – are needed to determine whether targeting MAP and serum sodium truly mitigates SAE and improves outcomes.

### 4.7. Future Research Directions and Conclusions

This work aims to accelerate clinical translation and enable precision intervention. Accordingly, we outline 5 follow-up directions. 1) Multicenter external validation – pool data from eICU, AmsterdamUMCdb, and large Asia-Pacific ICU registries. Assess model generalizability across ethnicities and care pathways, and explore the need for recalibration. 2) Dynamic multimodal fusion – integrate continuous MAP waveforms, cerebral oximetry, bedside EEG, and inflammatory/neural biomarkers within real-time EMRs. Train recurrent-neural-network or Transformer models to build early-warning systems that capture kidney–brain–immune dynamics. 3) Prospective adaptive trial – run a REMAP-CAP-style platform with 2 arms. Arm A: modest correction of mild hypernatremia. Arm B: individualized MAP targets derived from model-guided stratification. Primary end-points: 14-day SAE incidence and 28-day neurofunctional survival. 4) Decision-support embedding – integrate the nomogram-plus-SHAP module into a visual risk dashboard. Collaborate with ICU-information-system vendors, then evaluate clinician uptake and adherence via A/B testing. 5) Cost–benefit analysis – apply a Markov model to quantify the cost-effectiveness of model-driven electrolyte and blood-pressure interventions under varying resource scenarios. The resulting economic evidence will guide electrolyte management and BP targets across resource settings.^[33]^

## 5. Conclusion

Using the large MIMIC-IV database, we built and validated a streamlined risk model for sepsis-associated encephalopathy (SAE). The nomogram relies on just 4 readily available variables – age, SAPS II, serum sodium, and mean arterial pressure (MAP). It achieved robust discrimination, sound calibration, and a high clinical net benefit. The findings highlight mild hypernatremia and personalized MAP targets as modifiable treatment levers. SHAP analysis reveals kidney–brain–circulatory cross-talk underpinning SAE risk. Future work should test the model prospectively across multiple centers and embed it within clinical decision-support systems to advance precise, standardized brain protection in sepsis.

## Author contributions

**Conceptualization:** Zhiyang Zhang.

**Data curation:** Zhiyang Zhang, Dandan Li.

**Formal analysis:** Li Guo, Heling Zhao.

**Methodology:** Zhiyang Zhang, Ze Zhang, Limin Shen.

**Software:** Heling Zhao.

**Supervision:** Limin Shen.

**Visualization:** Li Guo.

**Writing – original draft:** Zhiyang Zhang, Ze Zhang, Li Guo.

**Writing – review & editing:** Zhiyang Zhang, Dandan Li, Limin Shen.

## Supplementary Material


