# Supplementary material for: Interpretable four-factor day-1 nomogram for predicting sepsis-associated encephalopathy in septic ICU patients with AKI: Development and internal validation in MIMIC-IV
Source: Medicine (Baltimore). 2026 Feb 13;105(7):e47726. doi: 10.1097/MD.0000000000047726 (PMC12908734; doi:10.1097/MD.0000000000047726)
Supplement: Supplementary file 1 [file medi-105-e47726-s001.docx]

**Supplementary Figure S1.** Internal validation of the SAE risk prediction model in the training cohort.

**
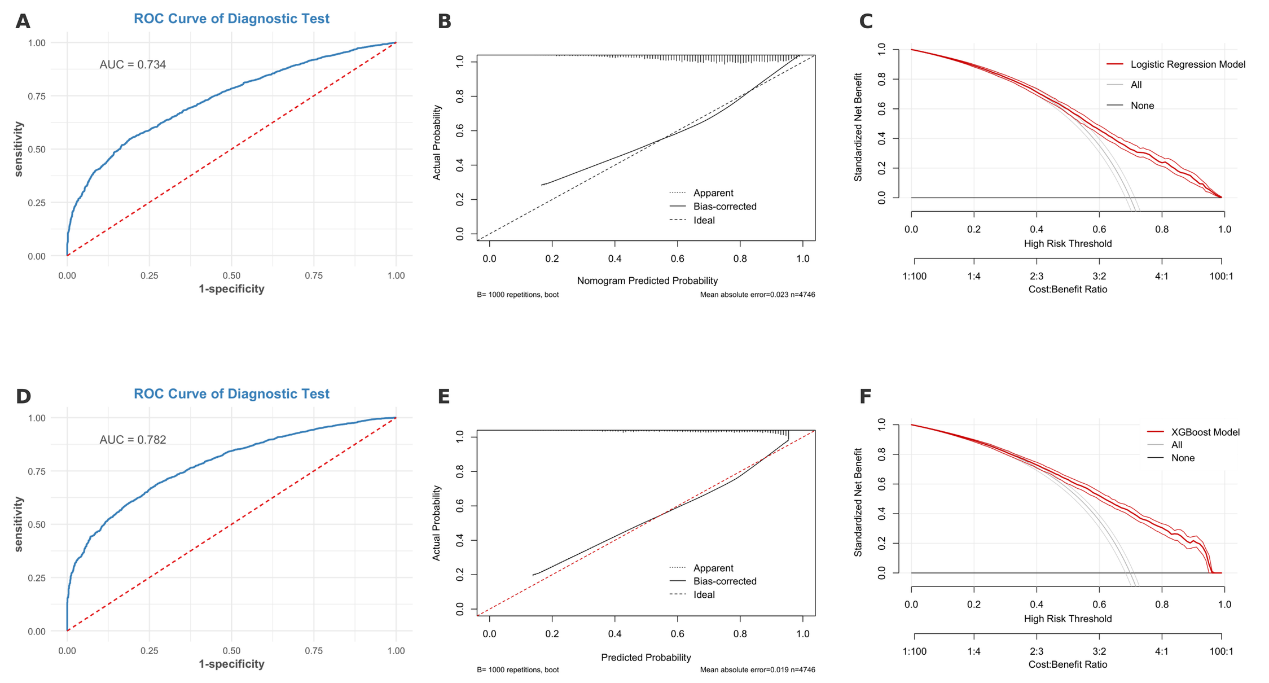
**

**Supplementary Figure S2.** Individual-level SHAP explanations and MAP-associated non-linear effect in the logistic regression model.

**
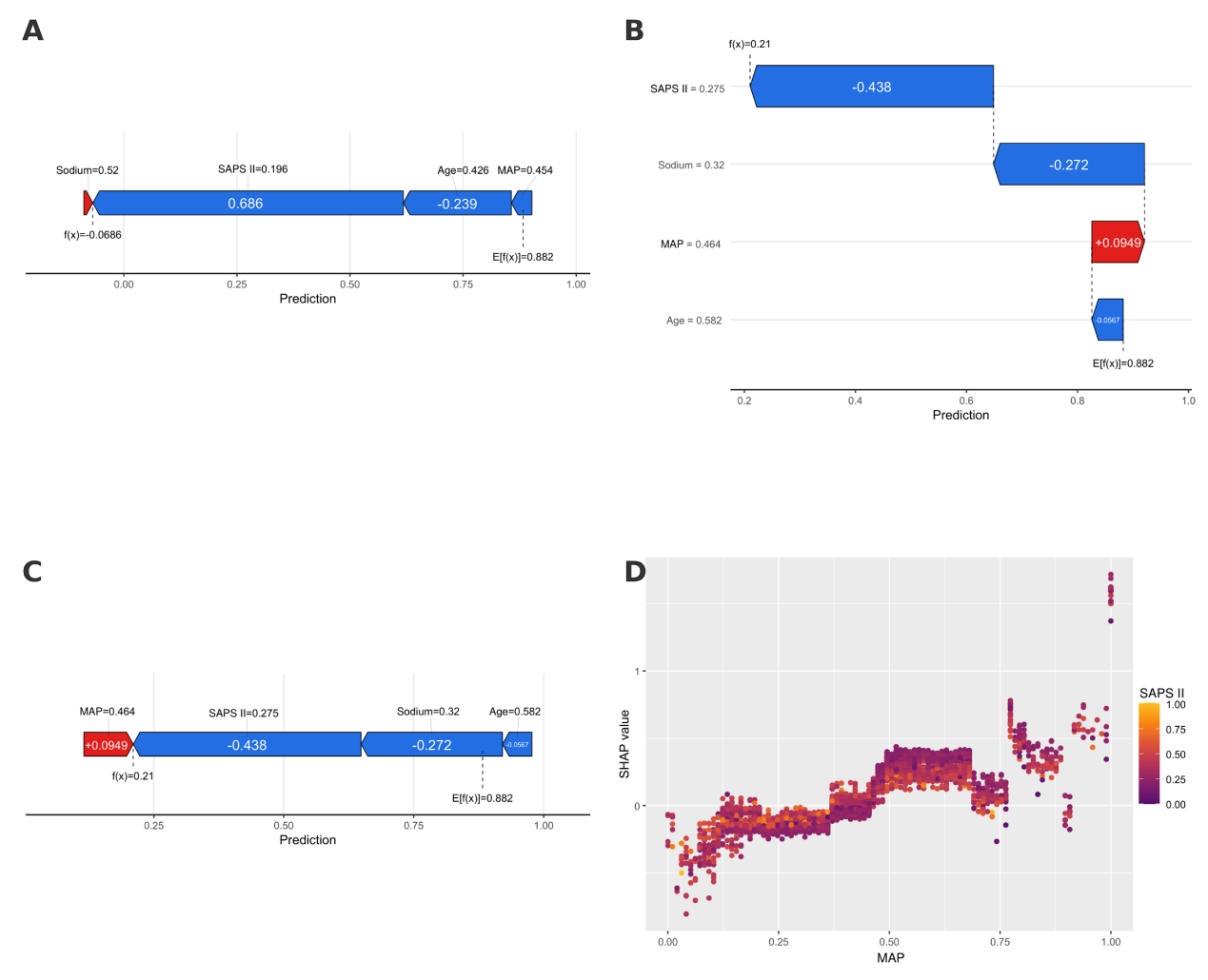
**

**Supplementary Table:**

**Supplementary Table S1. ICD-9/10 codes and exclusion-criteria list.**

**Exclusion of Patients with Primary Brain Injuries Based on ICD-9/10 Codes.**

Primary Brain Injury:

Traumatic Brain Injury (TBI): ICD-9 854.00, ICD-10 S06.9X0A

Ischemic Stroke: ICD-9 433.x1, ICD-10 I63.9

Hemorrhagic Stroke: ICD-9 431, ICD-10 I61.9

Epilepsy: ICD-9 345.x, ICD-10 G40.9

Intracranial Infection: ICD-9 324.x, ICD-10 G06.0

**Exclusion of Patients with Psychiatric and Neurological Disorders According to ICD-9/10 Codes.**

Psychiatric and Neurological Disorders

Major Depressive Disorder (MDD)

29634 - Major depressive episode, recurrent, with psychotic features

29635 - Major depressive episode, recurrent, partial or unspecified remission

29636 - Major depressive episode, recurrent, in full remission

Bipolar I Disorder

29640 - Bipolar I disorder, current manic episode, unspecified

29641 - Bipolar I disorder, current mild manic episode

29642 - Bipolar I disorder, current moderate manic episode

29643 - Bipolar I disorder, current severe manic episode without psychotic features

29644 - Bipolar I disorder, current severe manic episode with psychotic features

29650 - Bipolar I disorder, current depressive episode, unspecified

29651 - Bipolar I disorder, current mild depressive episode

29652 - Bipolar I disorder, current moderate depressive episode

Delusional Disorder

2971 - Delusional disorder

2972 - Paranoid state

Schizophrenia

29500 - Simple schizophrenia, unspecified

29501 - Simple schizophrenia, subacute

29502 - Simple schizophrenia, chronic

29510 - Disorganized schizophrenia, unspecified

29520 - Catatonic schizophrenia, unspecified

29530 - Paranoid schizophrenia, unspecified

Dementia

29410 - Dementia due to other conditions without behavioral disturbance

29411 - Dementia due to other conditions with behavioral disturbance

3310 - Alzheimer’s disease

33182 - Dementia with Lewy bodies

33119 - Other frontotemporal dementia

Other Neurological and Psychiatric Disorders

2980 - Depressive type psychosis

2981 - Excited type psychosis

2982 - Reactive confusion

2989 - Unspecified psychosis

**Exclusion of Patients with Long-term Alcohol or Drug Abuse Using ICD-9/10 Codes.**

Long-term Alcohol or Drug Abuse

Alcohol Dependence and Intoxication

30303 - Acute alcoholic intoxication in alcoholism, in remission

30390 - Other and unspecified alcohol dependence, unspecified

30391 - Other and unspecified alcohol dependence, continuous

30392 - Other and unspecified alcohol dependence, episodic

30393 - Other and unspecified alcohol dependence, in remission

Opioid Dependence

30400 - Opioid type dependence, unspecified

30401 - Opioid type dependence, continuous

30402 - Opioid type dependence, episodic

30403 - Opioid type dependence, in remission

Sedative, Hypnotic, or Anxiolytic Dependence

30410 - Sedative, hypnotic, or anxiolytic dependence, unspecified

30411 - Sedative, hypnotic, or anxiolytic dependence, continuous

30412 - Sedative, hypnotic, or anxiolytic dependence, episodic

30413 - Sedative, hypnotic, or anxiolytic dependence, in remission

Cocaine Dependence

30420 - Cocaine dependence, unspecified

30421 - Cocaine dependence, continuous

30422 - Cocaine dependence, episodic

30423 - Cocaine dependence, in remission

Cannabis Dependence

30430 - Cannabis dependence, unspecified

30431 - Cannabis dependence, continuous

30432 - Cannabis dependence, episodic

30433 - Cannabis dependence, in remission

Amphetamine and Other Psychostimulant Dependence

30440 - Amphetamine and other psychostimulant dependence, unspecified

30441 - Amphetamine and other psychostimulant dependence, continuous

30442 - Amphetamine and other psychostimulant dependence, episodic

30443 - Amphetamine and other psychostimulant dependence, in remission

Other Drug Dependence

30460 - Other specified drug dependence, unspecified

**Supplementary Table S2.** Number and Proportion of Missing Data for Each Factor in the Study Cohort (n = 6780)

| Factor Name | Missing Count | Missing Percentage |
| --- | --- | --- |
| Heart Rate | 2 | 0.029% |
| SBP | 26 | 0.38% |
| DBP | 26 | 0.38% |
| MAP | 2 | 0.029% |
| Temperature | 416 | 6.1% |
| Respiratory Rate | 4 | 0.058% |
| SpO_2_ | 3 | 0.04% |
| Hypertension | 5 | 0.074% |
| Diabetes | 5 | 0.074% |
| COPD | 5 | 0.074% |
| CKD | 5 | 0.074% |
| Cardiovascular Disease | 5 | 0.074% |
| BUN | 29 | 0.43% |
| Chloride | 27 | 0.40% |
| Scr | 28 | 0.41% |
| Sodium | 32 | 0.47% |
| Potassium | 43 | 0.63% |
| INR | 603 | 8.9% |
| PT | 603 | 8.9% |
| PTT | 632 | 9.3% |
| WBC | 45 | 0.66% |
| Platelet | 45 | 0.66% |
| Hemoglobin | 45 | 0.66% |
| Magnesium | 383 | 5.6% |
| Urine Output | 267 | 3.9% |

**Note**: Missing values were handled by excluding variables with more than 10% missing data. The remaining missing values (less than 10%) were addressed using multiple imputation with predictive mean matching (m = 5) for continuous variables and mode imputation for categorical variables. A subsequent sensitivity analysis will evaluate the impact of multiple imputation on the model's performance.

**Supplementary Table S3.** Across-imputation performance (m=5): AUC, accuracy, sensitivity, and specificity

| Data Set | AUC | Accuracy | Sensitivity | Specificity |
| --- | --- | --- | --- | --- |
| Data1 | 0.752 | 0.657 | 0.612 | 0.761 |
| Data2 | 0.752 | 0.642 | 0.573 | 0.802 |
| Data3 | 0.752 | 0.642 | 0.572 | 0.802 |
| Data4 | 0.750 | 0.670 | 0.644 | 0.730 |
| Data5 | 0.752 | 0.642 | 0.574 | 0.801 |

Note: Metrics were computed separately in each imputed dataset (m=5) and pooled using Rubin’s rules for reporting. Values in the table are per-imputation; pooled estimates are reported in the Results.

**Supplementary Table S4.** Univariate Logistic Regression Analysis of SAE Risk Factors.

|  |  |  |  |
| --- | --- | --- | --- |
|  | **β** | **Odds ratio(95%CI)** | ***P*** |
| Patient Characteristics |  |  |  |
| Age,(years) | 0.029 | 1.030(1.025-1.034) | <0.001 |
| Sex,n(%) | 0.401 | 1.494(1.313-1.699) | <0.001 |
| Race (Simplified) ,n(%) |  |  | 0.018 |
| B/A vs WHITE | -0.246 | 0.782(0.624-0.979) | 0.032 |
| OTHER vs WHITE | 0.077 | 1.080(0.785-1.487) | 0.636 |
| M/M vs WHITE | -0.225 | 0.799(0.617-1.034) | 0.008 |
| First Hospital Stay,n(%) | 0.285 | 1.330(1.147-1.543) | <0.001 |
| First ICU Stay,n(%) | 0.445 | 1.560(1.021-2.383) | 0.040 |
| Hospital Admission Frequency,n(%) |  |  | <0.001 |
| 2vs1 | -0.285 | 0.772(0.629-0.949) | 0.014 |
| 3 or mores times vs1 | 0.049 | 1.051(0.809-1.364) | 0.711 |
| AKI Stage,n(%) |  |  | <0.001 |
| AKI 2vs1 | -0.403 | 0.668(0.551-0.810) | <0.001 |
| AKI 3vs1 | -0.183 | 0.833(0.692-1.002) | 0.052 |
| Vital Signs |  |  |  |
| Heart Rate | 0.009 | 1.009 (1.005-1.012) | <0.001 |
| Respiratory Rate | 0.027 | 1.028 (1.017-1.039) | <0.001 |
| SBP,(mmHg) | 0.005 | 1.005 (1.002-1.007) | <0.001 |
| DBP,(mmHg) | 0.007 | 1.007 (1.003-1.010) | <0.001 |
| MAP,(mmHg) | 0.007 | 1.007 (1.004-1.011) | 0.001 |
| Temperature(℃) | 0.176 | 1.192 (1.102-1.290) | <0.001 |
| SpO_2_（%） | -0.030 | 0.970(0.953-0.988) | <0.001 |
| Comorbidities (n, %) |  |  |  |
| Hypertension,n(%) | -0.079 | 0.924 (0.805-1.060) | 0.259 |
| Diabetes,n(%) | -0.144 | 0.866 (0.748-1.003) | 0.055 |
| COPD,n(%) | -0.120 | 0.887 (0.745-1.056) | 0.176 |
| CKD,n(%) | -0.061 | 0.941 (0.818-1.082) | 0.393 |
| Cardiovascular Disease,n(%) | 0.013 | 1.013 (0.772-1.328) | 0.928 |
| Laboratory Parameters |  |  |  |
| BUN,(mmol/L) | 0.014 | 1.014 (1.010-1.017) | <0.001 |
| Chloride,(mmol/L) | -0.010 | 0.990(0.980-1.000) | 0.053 |
| Scr,(μmol/l) | -0.018 | 0.982 (0.945-1.020) | 0.342 |
| Sodium,(mmol/L) | 0.098 | 1.103 (1.086-1.121) | <0.001 |
| Potassium,(mmol/L) | -0.105 | 0.900 (0.820-0.988) | 0.027 |
| INR | 0.090 | 1.095 (1.012-1.188) | 0.031 |
| PT,(s) | 0.009 | 1.009 (1.000-1.018) | 0.050 |
| PTT,(s) | 0.004 | 1.004 (1.001-1.007) | 0.021 |
| WBC,(×10^9^/L) | 0.004 | 1.004 (0.996-1.012) | 0.351 |
| Platelet,(×10^9^/L) | 0.001 | 1.000 (0.999-1.000) | 0.279 |
| Hemoglobin,(g/L) | 0.021 | 1.021 (0.992-1.051) | 0.155 |
| Magnesium,(mmol/L) | -0.022 | 0.978 (0.872-1.097) | 0.706 |
| SAPS II | 0.063 | 1.065 (1.059-1.071) | <0.001 |
| Therapeutic Interventions |  |  |  |
| Ventilator,n(%) | -0.305 | 0.737 (0.651-0.835) | <0.001 |
| RRT,n(%) | -0.477 | 0.621 (0.477-0.808) | <0.001 |
| Sedative,n(%) | 0.099 | 1.104 (0.973-1.253) | 0.123 |
| Analgesic,n(%) | -0.333 | 0.717 (0.630-0.816) | <0.001 |
| H_2_ Antagonist,n(%) | -0.266 | 0.767 (0.661-0.888) | <0.001 |
| Heparin,n(%) | -0.399 | 0.671(0.589-0.765) | <0.001 |
| Diuretic,n(%) | -0.244 | 0.799 (0.706-0.905) | <0.001 |
| Vasoactive Agent,n(%) | 0.104 | 1.110(0.980-1.258) | 0.101 |
| Renal and Ventilation Parameters |  |  |  |
| Total Input ,n(%) |  |  | 0.126 |
| Medium vs Low | 0.072 | 1.075 (0.900-1.283) | 0.428 |
| High vs Low | -0.083 | 0.921 (0.790-1.072) | 0.287 |
| Total Output,n(%) |  |  | <0.001 |
| Medium vs Low | 0.631 | 1.880 (1.578-2.241) | <0.001 |
| High vs Low | 0.443 | 1.558 (1.344-1.806) | <0.001 |
| Fluid Balance,n(%) |  |  | 0.049 |
| Medium vs Low | -0.011 | 0.989 (0.828-1.181) | 0.902 |
| High vs Low | -0.161 | 0.851 (0.730-0.992) | 0.040 |
| Urine Output,n(%) |  |  | <0.001 |
| Medium vs Low | 0.647 | 1.909 (1.597-2.283) | <0.001 |
| High vs Low | 0.317 | 1.373 (1.184-1.593) | <0.001 |

SAE, sepsis-associated encephalopathy; MAP, mean arterial pressure; SBP, systolic blood pressure; DBP, diastolic blood pressure; SpO₂, peripheral oxygen saturation; BUN, blood urea nitrogen; Scr, serum creatinine; WBC, white blood cells; COPD, chronic obstructive pulmonary disease; CKD, chronic kidney disease; INR, international normalized ratio; PT, prothrombin time; PTT, partial thromboplastin time; SAPS II, Simplified Acute Physiology Score II; RRT, renal replacement therapy; B/A, Black or African-American; M/M, Mixed or multiracial. Odds ratios are reported relative to the reference category for each categorical variable. Input, output, urine output, and fluid balance were categorized as Low (<1500 mL), Medium (1500–2000 mL), and High (>2000 mL), based on totals from the first 24 h in ICU. Ventilator and RRT refer to initiation within the first 24 h.

**Supplementary Table S5.** Final logistic model equation and coefficients (original clinical units; ORs reported per clinically meaningful increments).

| **Predictor** | **Unit for OR** | **β (log-odds)** | **SE(β)** | **OR (per increment)** | **95% CI for OR** | **P value** |
| --- | --- | --- | --- | --- | --- | --- |
| Age | per 10 years | 0.012 | 0.00254 | 1.127 | 1.072–1.184 | <0.001 |
| MAP | per 5 mmHg | 0.013 | 0.00224 | 1.067 | 1.046–1.093 | <0.001 |
| Sodium | per 5 mmol/L | 0.097 | 0.00902 | 1.625 | 1.490–1.778 | <0.001 |
| SAPS II | per 10 points | 0.060 | 0.00313 | 1.825 | 1.708–1.931 | <0.001 |
| **Intercept (β₀)** | — | **β₀** | SE(β₀) | — | — | — |

Note: Model equation: logit(p) = β₀ + β₁·(Age/10) + β₂·(MAP/5) + β₃·(Sodium/5) + β₄·(SAPS II/10). Coefficients (β) and standard errors (SE) are estimated per 1-unit increase in the original clinical scales (Age: years; MAP: mmHg; Sodium: mmol/L; SAPS II: points). Per-increment odds ratios are reported as OR = exp(β × increment), and 95% confidence intervals as exp((β ± 1.96·SE) × increment), with increments fixed at 10 years (Age), 5 mmHg (MAP), 5 mmol/L (Sodium), and 10 points (SAPS II). The intercept (β₀) and its SE are available in the code repository / upon request.
